# Epidemiological Insights Into Relocation in Older Age From the Second Longitudinal Study of Aging (LSOA II): A Cross‐Sectional Study

**DOI:** 10.1155/sci5/5513056

**Published:** 2026-09-02

**Authors:** Maximilian Andreas Storz, Alvaro Luis Ronco

**Affiliations:** ^1^ Department of Internal Medicine II, Centre for Complementary Medicine, Medical Center–University of Freiburg, Faculty of Medicine, University of Freiburg, Freiburg, Germany, uni-freiburg.de; ^2^ Department of Medical Records, National Cancer Institute of Uruguay, Juanicó 3265, Montevideo 11600, Uruguay

**Keywords:** aging, epidemiology, health, moving, older age, relocation

## Abstract

**Background:**

As the global population ages as a whole, interest in the “place” dimension of aging is growing. Previous studies on relocation in older age were often qualitative in nature and based on small samples. Large‐scale epidemiological studies that specifically account for nonmovers are scarce but may contribute to a better understanding of older people’s reasoning about relocation.

**Methods:**

Data from the Second Longitudinal Study of Aging–Wave II, a U.S. population–based survey from 1997 to 1998, was analyzed to explore the most common reasons for moving in adults aged ≥ 70 years. The data were analyzed with a cross‐sectional analytical approach. The predicted probability of moving depending on age and sociodemographic factors was estimated based on survey‐weighted multivariable logistic regression. Moving/relocation was self‐reported and covered a preceding 2‐year period; reasons for moving implied a permanent relocation.

**Results:**

The weighted percentage of participants reporting relocation within the last 2 years was 10.83% (9.81–11.95). The predicted probability of moving increased with higher age and doubled from 10% to 20% when comparing those aged 70 years and 90 years. Participants reporting fair/poor overall health were significantly more likely to have moved than those participants with excellent health (contrast: +7% (95% CI: 4.1%–9%), *p* < 0.001). A similar contrast was found when comparing married participants with widowed/divorced participants (+6.8% (95% CI: 3%–10%), *p* = 0.001). Deteriorating health and the desire to go to a smaller place were the most frequently reported reasons for moving with 35.67% (31.98–39.53) and 18.71% (15.29–22.70), respectively. More than 10% of participants who relocated indicated a desire to live closer to their child/children.

**Conclusions:**

Results from this epidemiological analysis corroborate motivations for relocation identified in previous interview‐based qualitative studies. The estimated marginal predicted probabilities provide new insights into the complex dynamics of relocation in older age.

## 1. Introduction

Relocation in older age and residential mobility have been important topics in gerontological and population research for several decades [1]. Reasoning about aging in place and relocation in older age is an ambivalent and complex matter [2]. Commonly defined as remaining in one’s familiar environment, “aging in place” is often associated with independence and autonomy [3, 4]. Relocation, on the other hand, is more broadly defined as a movement from one environment to another [5]. Numerous studies investigated reasons for and barriers to relocation and aging in place in older age, respectively [1, 2].

Factors associated with aging in place include strong ties to children, strong social capital, housing wealth, neighborhood satisfaction, and homeownership status [6]. Factors associated with relocation include the wish for a smaller, cheaper, or obstacle‐free flat, as well as the desire for changing neighborhood [7]. Low social class and living in rural areas are also associated with a higher probability of moving in older age [8]. The same applies to family factors, for example, the loss of a partner [7] and chronic health conditions [9].

As the global population as a whole ages, interest in the “place” dimension of aging is simultaneously growing [6]. Aging in place is preferred by the majority of adults but is also associated with challenges and barriers [10, 11]. That said, research concerning older people’s reasoning about where they want to grow old is an ongoing topic that needs to be further investigated [1]. Previous studies in this field were mainly interview‐based, small, and focused on qualitative components and interrogations. However, large‐scale, population‐based epidemiological studies that employ a complex survey design in this field are scarce, and only a few studies specifically took so‐called “non‐movers” into account [2]. The latter appears of utmost importance to estimate predicted probabilities for moving, which is not possible without a “non‐moving” reference group. Considering these gaps in the literature, we revisited data from the Second Longitudinal Study of Aging (LSOA II)–Wave II, a U.S. population–based survey with a complex sampling design [12, 13]. Data from this survey were analyzed by Pope and Kang to compare proactive and reactive movers [14].

Beyond the identification of reasons for moving in adults aged 70 years or older, we aimed to estimate the predicted prevalence of moving depending on age and other sociodemographic factors using an epidemiological approach at the national level by specifically taking nonmovers into account. By extending the work of Pope and Kang and by exploring historical perspectives, the rationale was to gain additional descriptive insights into moving in older age at the population level from an epidemiological perspective based on survey data.

## 2. Methods

### 2.1. General Study Information

The Longitudinal Studies of Aging (LSOAs) are a multicohort study of non‐institutionalized U.S.‐based individuals aged 70 years or older [12, 13]. The main study goal was to measure changes in health, functional status, living arrangements, and health service utilization. Four major surveys were originally included: the 1984 Supplement on Aging, the 1984–1990 Longitudinal Study of Aging (LSOA), the 1994 Second Supplement on Aging (SOA II), and the 1994–2000 LSOA II. The 1994 SOA II served as the baseline for the LSOA II. The latter was conducted 10 years after the original LSOA in a joint effort of the National Center for Health Statistics (NCHS) and the NIA (National Institute on Aging). The sample included *n* = 9447 participants; all sample persons selected were at least 70 years old in 1995. Data collection for the LSOA II took place between May 1997 and May 1998.

### 2.2. Ethical Approval

The SOA II was conducted as a supplement to the 1994 National Health Interview Survey (NHIS). The NHIS is a public health surveillance activity excluded from the regulatory requirements of 45 CFR 46 [15]; procedures and protocols were reviewed and approved by the NCHS Ethics Review Board to protect the rights and welfare of participants [16]. The LSOA II was a supplement to the annual NHIS in 1994 and also embedded in the aforementioned legal framework.

### 2.3. Survey Design

The LSOA II Wave 2 dataset is freely available online and includes data from various sources merged into a single data file [12, 13]. Data were collected in 1997 and 1998. The LSOA Wave 2 was conducted by telephone using computer‐assisted interviews. Wave 2 differentiated between a survivor questionnaire and a decedent version of the questionnaire, which was completed with the help of proxy respondents. For this study, we restricted ourselves to the survivor questionnaire. Except for the baseline data (person data for sample persons, locations 1–200 in the survivor data codebook), we restricted ourselves to LSOA II Wave 2 survivor variables (location 1827 or greater in the survivor data codebook). LSOA II uses a multistage sample design, which represents the non‐institutionalized civilian U.S. population. Appropriate sample weights were used following the recommended procedure (method 1, page 49) described in the data user guide [12, 13].

### 2.4. History of Relocation/Moving

The main outcome/dependent variable of interest was relocation/moving since the baseline SOA II interview (roughly 2 years prior to the LSOA II Wave 2). Participants were asked whether they moved since the last interview with the following options for reply: “Yes,” “no,” “don’t know” (codebook location 1867 in the survivor data codebook). While the question was not defined in greater detail, the answer options for moving (detailed below) largely implied a permanent relocation. Participants who replied with “don’t know” were not considered (*n* = 9 participants). Subsequently, participants were asked when they moved and why. The LSOA II Wave two interview included numerous prespecified reasons, which read as follows: “sample person’s health deteriorated,” “sample person’s health improved,” “spouse’s health deteriorated,” “spouse’s health improved,” “to go to a different climate,” “sample person moved to a nursing home,” “spouse moved to a nursing home,” “spouse died,” “divorced, separated from spouse,” “live close to child/children,” “to live with child/children,” “live with or closer to relative,” “change in people live, help sample person,” “go to a smaller house/apartment,” “for financial reasons,” “structural limits of old place,” “go to a better, safer neighborhood,” go to retirement home, community,” “get closer to a health facility,” “other reasons.” Additionally, we analyzed the month/season of moving [1, 2]. All variables described in this paragraph were LSOA II Wave 2‐specific.

### 2.5. Covariables

Covariables that have been associated with relocation in older age in earlier studies included sex (male or female), marital status (married; widowed/divorced/separated; unmarried/never married; unknown), race/ethnicity (White; Black; Other), educational level (elementary; high school; high school graduate; college; college graduate; postcollege) and income (less than $20,000 or $20,000 and above) [1, 2, 17–19]. Age was considered, as well (continuous variable). Covariables were selected based on the previous literature and based on availability [1, 2, 17–19]. For example, data on neighborhood safety, an important covariable when it comes to relocation in older age, was not readily available and could thus not be included [7]. All variables described in this paragraph were LSOA II Wave 2‐specific, except for race/ethnicity, education, and income.

### 2.6. Statistics

Data were downloaded from the official website and imported into Stata 19 [20]. Stata’s survey commands (including “.svyset” and “.svy:”) were used throughout the analysis. In a first step, we excluded participants with missing data on any variable of interest. We thereby defined a cohort with a complete dataset for various preselected covariates and an available history of moving/relocation since the baseline SOA II interview. Following the Strengthening the Reporting of Observational Studies in Epidemiology (STROBE) guidelines for reporting observational studies, we provided a step‐by‐step exclusion flowchart [21]. No data were imputed. Participants were mainly excluded for missing marital status data, a sociodemographic variable that was deemed to be of high relevance for the current study.

As for the descriptive statistics, we provided weighted percentages/proportions (with their 95% CI in parenthesis) and means (with their 95% CI in parenthesis). Associations between a history of relocation and sociodemographic data were examined using Stata’s Rao–Scott Chi‐square test (in the case of categorical variables) or univariate regression (in the case of age). In part I of the analysis, we subsequently analyzed the main reasons participants provided for the relocation since the baseline SOA II interview. Data were visualized using a bar chart with survey weights. In part II of the analysis, we estimated marginal predicted probabilities for moving since the baseline SOA II interview. For this, we constructed a multivariate logistic regression model and used Stata’s postestimation commands, margin, and marginsplot [22]. The model included the following independent variables: sex, age, marital status, race/ethnicity, educational level, income, and health status. Relocation since the baseline SOA II interview was the dependent variable (binary, categorized into yes or no). For all analyses, a *p* value < 0.05 was used as a cutoff for statistical significance.

## 3. Results

The final sample included *n* = 6091 individuals aged 70 years or older. A participant inclusion flowchart is provided in the supporting data (Supporting Figure 1). In total, *n* = 3356 participants had to be excluded for missing data, thereof *n* = 2406 for missing relocation status due to nonparticipation in wave 2.

Table 1 displays the sample’s characteristics. Sociodemographic data are shown separately for those who moved since the baseline SOA II interview (*n* = 663) and for those participants who did not move since the last interview (*n* = 5428). The weighted percentage of participants reporting relocation was 10.83% (9.81–11.95). A history of relocation was not independent from sex, marital and health status, and income as well as the participant’s age. Those who moved since the baseline SOA II interview tended to be older and reported fair/poor overall health more frequently.

**TABLE 1:** Sample characteristics: weighted percentages based on *n* = 6091 unweighted observations.

Variable	Moved since the last interview (*n* = 663)			Did not move since the last interview (*n* = 5428)			*p* value
	Weighted percentage	Lower 95% CI	Upper 95% CI	Weighted percentage	Lower 95% CI	Upper 95% CI	
Sex							**0.001**
Male	32.26%	28.88	35.84	39.07%	37.78	40.37	
Female	67.74%	64.16	71.12	60.93%	59.63	62.22	
Marital status							**< 0.001**
Married	25.74%	21.90	30.00	41.97%	40.37	43.59	
Widowed/divorced/separated	58.16%	53.94	62.27	43.50%	41.8	45.21	
Unmarried/never married	10.85%	8.52	13.72	7.48%	6.76	08.28	
Unknown	5.24%	3.86	7.09	7.05%	6.44	07.71	
Race/ethnicity							0.305
White	92.15%	89.92	93.93	90.69%	89.32	91.90	
Black	5.76%	4.25	7.77	7.52%	6.53	8.66	
Other	2.09%	1.22	3.55	1.79%	1.16	2.74	
Educational level							0.050
1–8 years (elementary)	25.47%	21.98	29.31	21.46%	20.04	22.96	
9–11 years (high school)	14.00%	11.21	17.34	15.29%	14.09	16.58	
12 years (high school graduate)	33.48%	29.46	37.77	34.95%	33.47	36.46	
1–3 years (college)	14.37%	12.10	16.97	14.56%	13.55	15.63	
4 years (college graduate)	9.12%	6.90	11.96	7.74%	6.97	8.59	
5 or more years (postcollege)	3.57%	2.36	5.36	6.00%	5.29	6.79	
Income							**0.007**
Less than $20,000	54.77%	50.28	59.19	47.86%	46.06	49.67	
$20,000 or more	40.64%	36.08	45.36	47.94%	46.15	49.74	
Unknown	4.59%	2.98	7.00	4.20%	3.50	5.02	
Self‐rated overall health status							**< 0.001**
Excellent/very good	31.35%	27.85	35.08	40.87%	39.51	42.25	
Good	29.70%	26.11	33.57	33.64%	32.28	35.02	
Fair/poor	38.94%	34.75	43.30	25.49%	24.19	26.84	
Age	78.72	78.23	79.20	76.51	76.34	76.68	**< 0.001**

*Note:* Age shown as mean (95% CI). Categorical variables shown as weighted percentages (95% CI). All *p* value are based on Stata’s design‐adjusted Rao–Scott test except for age, which is based on univariate regression analysis. Significant *p* values are shown in bold.

**FIGURE 1:**
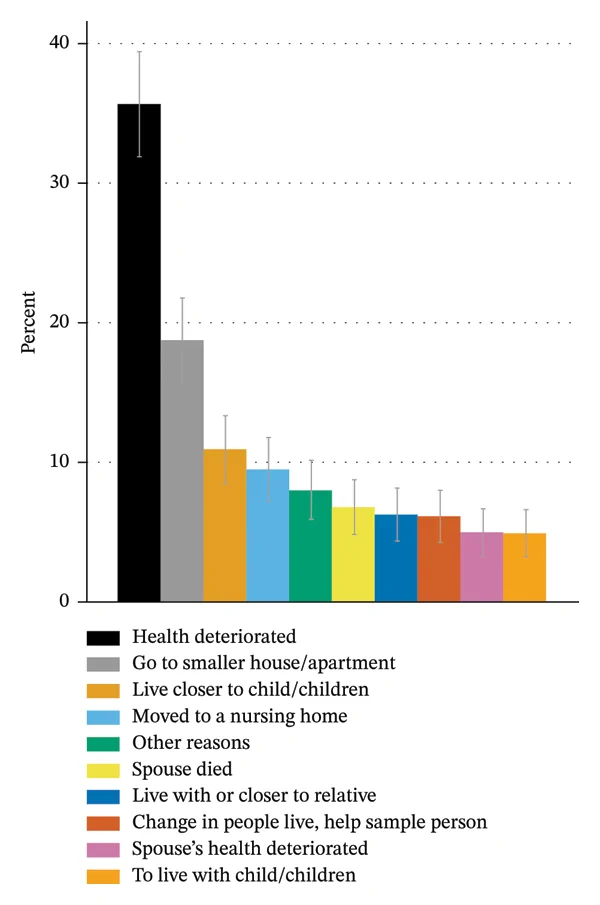
Bar chart depicting the top 10 reasons for participants’ relocation since their last interview in descending frequency (based on *n* = 663 observations).

**FIGURE 2:**
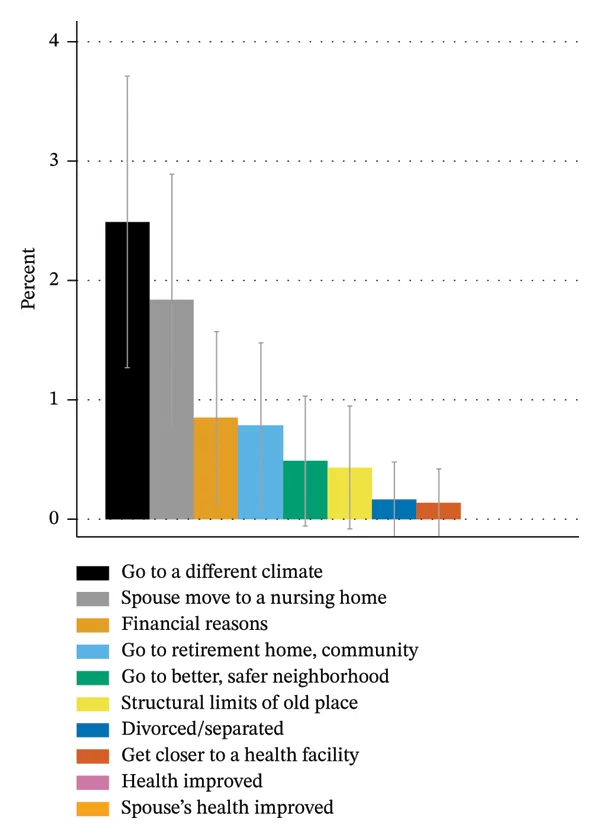
Bar chart depicting rare reasons for participants’ relocation since their last interview in descending frequency (based on *n* = 663 observations).

Figure 1 displays the top 10 reasons that participants reported for relocation since the baseline SOA II interview in descending frequency. Deteriorating health and the desire to go to a smaller place/apartment were the most frequently reported reasons, with 35.67% (31.98–39.53) and 18.71% (15.29–22.70), respectively. More than 10% of participants who relocated since the baseline SOA II interview indicated a desire to live closer to their child/children. Participants rarely relocated because their health improved. Less common reasons (prevalence < 5% in the sample) are displayed in Figure 2.

**FIGURE 3:**
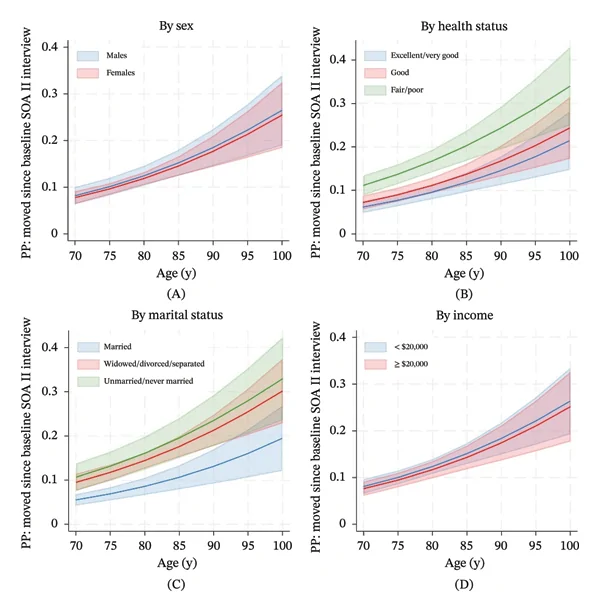
Marginal predicted probabilities for moving since the last LSOA II interview based on a multivariate logistic regression model (based on *n* = 5444 unweighted observations). Based on a multivariate regression model adjusting for sex, age, marital status, race/ethnicity, educational level, income, and health status. The model was statistically significant: *F* (14, 173) = 10.53, *p* < 0.001. No significant overall contrasts between females and males were found in panel A. Those reporting fair/poor overall health (panel B) were significantly more likely to move than those with excellent health (contrast: +6.5% (95% CI: 4.1%–9%), *p* < 0.001). The contrast between married participants and those who were never married/unmarried was significant as well (contrast: +6.8% (95% CI: 3%–10%), *p* = 0.001) as shown in panel C. A comparable picture was found when contrasting married participants with widowed/divorced participants (contrast: +5.3% (95% CI: 3%–7.6%), *p* < 0.001). Panel D showed no significant contrasts when comparing the two prespecified income categories. PP = predicted probability.

Figure 3 shows marginal predicted probabilities for moving since the baseline SOA II interview based on a multivariate logistic regression model covering *n* = 5444 unweighted observations. This model included the following covariates: sex, age, marital status, race/ethnicity, educational level, income, and health status. No significant overall contrasts between females and males were found (panel A). Those reporting fair/poor overall health (panel B) were significantly more likely to have moved than those with excellent health (contrast: +6.5% (95% CI: 4.1%–9%), *p* < 0.001). The contrast between married participants and those who were never married/unmarried was also significant (contrast: +6.8% (95% CI: 3%–10%), *p* = 0.001) as shown in panel C. A similar contrast was found when contrasting married participants with widowed/divorced participants (contrast: +5.3% (95% CI: 3%–7.6%), *p* < 0.001). Panel D shows no significant contrasts when comparing the two prespecified income categories.

Additional diagnostic plots for a deeper understanding of the analyzed data are displayed in Supporting Figures 2–5. Supporting Figure 2 shows the number of reasons participants provided for moving. Approximately 5% of participants (*n* = 35) provided no reason at all, whereas 79.17% (*n* = 524) of participants provided one reason. A total of 91 participants (13.28%) provided two reasons, highlighting that moving is—in several instances—a composite decision influenced by various external factors.

Supporting Figure 3 includes a coefficient plot displaying the regression coefficients underlying Figure 2 as well as a comparison between the crude (age only) logistic regression model and the final fully adjusted model. We also investigated whether the month of moving showed a particular distribution. While there was a tendency to move in the summer months (e.g., 9.76% of the sample moved in July and 10.47% moved in August), a higher share of participants also moved in December (8.37%) and January (7.98%) (Supporting Figure 4). As shown in Supporting Figure 5, the number of individuals moving in the winter months slightly increased with older age, whereas the number of individuals moving in the summer months decreased with higher age. At this stage, we cannot explain this observed phenomenon. None of the main examined reasons for moving was associated with moving in summer or winter in bivariate analyses, respectively (Supporting Table 2).

## 4. Discussion

This study used LSOA II–Wave II data to estimate the predicted prevalence of moving depending on age and other sociodemographic factors in U.S. adults aged 70 years or older at the national level. Results may be briefly summarized as follows: Approximately 11% of participants relocated within the last 2 years; the predicted probability of moving increased with higher age. Those reporting fair/poor overall health were significantly more likely to move than those with excellent health. Deteriorating health and the desire to go to a smaller place/apartment were the most frequently reported reasons for relocation with 35.67% (31.98–39.53) and 18.71% (15.29–22.70), respectively. Participants rarely relocated because their health improved.

Results from this study largely align with previous studies investigating reasons for and barriers to relocation in older age. In a hallmark study in the field, Swedish and German researchers used a cross‐national qualitative design to explore beliefs and reflection upon relocation and aging in place in 80 older adults [2]. Most participants expressed the wish to age in place, with maintenance of autonomy and privacy as the primary goals. Considerable attempts to avoid loneliness were undertaken, as well. As in our study, major changes in health conditions that may cause significant changes to everyday life were commonly expressed in the Löfqvist study. Having progressive dementia or a stroke was unambiguously considered to make it impossible to continue living an independent life at home [2]. Unsurprisingly, deteriorating health was by far the most common reason for relocation expressed by adults in the LSOA II–Wave 2.

The second most frequent reason for moving in this epidemiological analysis was the desire to relocate to a smaller place/apartment. This trend has been previously reported in a Berlin‐based study from Germany [7]. Depending on existing comorbidities, older people may experience severe difficulties in houses with poor accessibility and a lack of adaptation for functional limitations [23]. Downsizing and having fewer commodities is often perceived as an act of liberation in older adults, resulting in a higher quality of life [24]. Some authors go as far as describing downsizing as a “*part of a life-long normative process of change and adjustment as older adults take on new roles in their new places, some expected and others not*” [25]. The fact that almost one out of five participants in the present survey identified the desire to go to a smaller house/apartment as the main reason for moving highlights the importance of downsizing in the life course. These findings may have important implications from a public health policy perspective, including the need for improved information provision about housing choices in later life (including practical aspects of downsizing) and a potential need for specialized services to assist older people in the downsizing process when anticipating relocation [26]. The latter may include advice on forward planning for care and housing needs as well as financial advice in the process.

The present findings further corroborate results of a 2021 study by Li et al. that was based on 2017 American Housing Survey data [27]. The authors found that those who moved tended to be those with lower incomes, those with higher housing cost burdens, and those who lived alone. The top reasons for moving, however, differed from our study: living closer to family members (41%), better neighborhoods (29%), and reducing housing costs (25%) were the most frequently reported reasons. Of note, the present study focused on adults aged 70 years or older (mean age 78.72 years), whereas Li et al. investigated adults aged ≥ 60 years [27]. Another reason that may explain the differences is the time gap (20 years) between the two surveys. Sociodemographic and societal changes may have led to other priorities in older adults. Perceived neighborhood safety, for instance, has profound impacts on older adults and their health [28]. Reviews emphasized that neighborhood safety appears nowadays most relevant to mental health and physical function [29], and it is likely that in current times a higher share of older adults would indicate this in surveys about relocation in older age. Contrasting our results to the 2021 analysis by Li et al. [27], we found noticeable similarities and contrasts. Li et al. reported that movers tended to live alone and had lower incomes. The first observation is also supported by the herein‐presented data, while the second observation was not applicable to our findings, as no relevant contrasts were found between the two examined income groups.

This study has several strengths and weaknesses that warrant further consideration. A major weakness is that the data stem from the last century (1997). However, to the best of our knowledge, this dataset has not been fully exploited in the past with regard to this particular outcome. Historical perspectives may serve as a basis for foresight of population reactions and trend analyses and may help evolve or validate trends in population behaviors [30]. Not analyzing these data to the fullest would result in a missed opportunity, particularly because national, epidemiological, survey‐based data in this field is still scarce. Data on comorbidities were not included except for self‐perceived health, which may also be an additional limitation. Another limitation concerns survivor bias. Inherent to the data source, only participants who were alive at the time of the interview were considered. Many participants were excluded due to missing marital status data. The cross‐sectional analytical approach did not permit any causal inferences. As for the strengths, the study is characterized by a fairly large sample size, and the multivariate models also take nonmovers into account. This study also extends previous findings by Pope and Kang [14], who used the same dataset to compare reasons for moving in those who moved proactively with those who moved reactively. Our study goals differed in a way that we focused on multivariate regression models that explicitly took survey weights and nonmovers into account. We also acknowledge that reasons for moving in the LSOA II dataset were precoded. Questions about motivations for relocation were not open‐ended [14], which may have introduced various biases to the data and could be seen as a limitation. Finally, the time span between the interview and the baseline interview was only 2 years, which potentially contributed to a limited number of movers.

## 5. Conclusions

Results from this epidemiological analysis corroborate motivations for relocation identified in previous studies and reemphasize the complexity of relocation in older age. The estimated marginal predicted probabilities in this study highlight the complex dynamics of aging, the predicted probability of moving depending on sociodemographic factors, and the resulting challenges for the U.S. population that is increasingly characterized by a longer life and shorter health span. While somewhat dated, the herein presented data may still be used by policymakers, gerontologists, and community planners in conjunction with other data sources discussed in this article to create age‐inclusive communities and to learn more about the needs of movers in older age.

## Funding

The authors received no financial support/funding.

Open access funding was enabled and organized by Projekt DEAL.

## Ethics Statement

The present study is negligible risk research that involves existing collections of freely available and non‐identifiable data about human beings. Research was performed in accordance with the Declaration of Helsinki. The National Health Interview Survey (NHIS) is a public health surveillance activity excluded from the regulatory requirements of 45 CFR 46; procedures and protocols are reviewed and approved by the NCHS Ethics Review Board to protect the rights and welfare of participants. The LSOA II was a supplement to the annual National Health Interview Survey in 1994 and embedded in the aforementioned legal framework. The local ethical committee of the University of Freiburg exempted the analysis from a secondary institutional review board submission.

## Conflicts of Interest

The authors declare no conflicts of interest.

## Supporting Information

Additional supporting information can be found online in the Supporting Information section.

## Supporting information


**Supporting Information** Supporting Table 1: STROBE Checklist. STROBE Statement—Checklist of items that should be included in reports of cross‐sectional studies. Supporting Table 2: Bivariate associations between specific reasons for moving and the time of moving (moving in the winter months [top] versus moving in the summer months [bottom]). Based on a subsample of movers with *n* = 550 participants). Supporting Figure 1: Participant inclusion flowchart. The final sample comprised *n* = 6901 participants, thereof *n* = 663 who moved since the LSOAII baseline interview and who were analyzed in part I of the study. For part II (analysis of marginal predicted probabilities), we excluded individuals with unknown marital status and unknown income (but otherwise available data), resulting in a final sample size of *n* = 5444 participants. Supporting Figure 2: Number of reasons participants provided for moving. Based on *n* = 663 participants. 79.17% (*n* = 524) of participants provided one reason, while 5.20% of participants (*n* = 35) provided no reason at all. A total of 91 participants (13.28%) provided two reasons. Supporting Figure 3: Coefficient plot (left side) displaying regression coefficients for the regression model underlying Figure 2. The right side compares the fully adjusted logistic regression model in red color (adjusted for sex, age, marital status, race/ethnicity, educational level, income, and health status) with a crude logistic regression model (age only) in blue color. Supporting Figure 4: Bar chart displaying the month of moving in *n* = 663 participants. Supporting Figure 5: Marginal predicted probabilities for moving in the winter months (panel A) or in the summer months (panel B) since the last LSOA II interview based on a multivariate logistic regression model (based on *n* = 663 unweighted observations who indicated relocation).

## Data Availability

Data are publicly available online (https://archive.cdc.gov/www_cdc_gov/nchs/lsoa/lsoa2.htm). The datasets used and analyzed during the current study are available from the corresponding author on reasonable request.
