# Supplementary material for: Epidemiological Insights Into Relocation in Older Age From the Second Longitudinal Study of Aging (LSOA II): A Cross‐Sectional Study
Source: Scientifica (Cairo). 2026 Sep 2;2026:5513056. doi: 10.1155/sci5/5513056 (PMC13535850; doi:10.1155/sci5/5513056)
Supplement: Supplementary file 1 [file SCI5-2026-5513056-s001.docx]

# Supplementary Data

# Supplementary Tables

## Supplementary Table 1: STROBE Checklist

STROBE Statement—Checklist of items that should be included in reports of ***cross-sectional studies***

|  | Item No | Recommendation |
| --- | --- | --- |
| **Title and abstract** | 1 | (*a*) Indicate the study’s design with a commonly used term in the title or the abstract  *-> indicated in the abstract under the subheading “Methods”* |
|  |  | (*b*) Provide in the abstract an informative and balanced summary of what was done and what was found  *-> Page 2, abstract, results emphasizes “The weighted percentage of participants reporting relocation within the last two years was 10.83% (9.81-11.95). The predicted prevalence of moving increased with higher age; no significant contrasts were found between females and males. Those reporting fair/poor overall health were significantly more likely to move than those with excellent health. A similar picture was found when contrasting married participants with widowed/divorced participants. Deteriorating health and the desire to go to a smaller place were the most frequently reported reasons for moving with 35.67% (31.98-39.53) and 18.71% (15.29-22.70), respectively. More than 10% of participants who relocated indicated a desire to live closer to their child/children. Participants rarely relocated because their health improved.”* |
| Introduction | | |
| Background/rationale | 2 | Explain the scientific background and rationale for the investigation being reported  *-> Page 3 summarizes the lack of large, epidemiological studies in the field of gerontology. The major rationale for this study is the lack of studies that investigation motivations for and barriers to relocation in older age.* |
| Objectives | 3 | State specific objectives, including any prespecified hypotheses  *-> Page 3, last paragraph before methods. “The rationale was twofold: I) historical perspectives may serve as a basis for foresight of population reactions and trend analyses, and may help evolve or validate trends in population behaviors (Dexter, 2006); II) to gain additional insights into moving in older age on a population level from an epidemiological perspective based on the consideration of non-movers and large-population based survey data.”* |
| Methods | | |
| Study design | 4 | Present key elements of study design early in the paper  *-> Page 3, methods: “The Longitudinal Studies of Aging (LSOAs) are a multicohort study of US-based individuals aged 70 years or older (Centers for Disease Control and Prevention, 2025; National Center for Health Statistics, 2002). The study goal was to measure changes in the health, functional status, living arrangements and health service utilization. Four major surveys were originally included: the 1984 Supplement on Aging, the 1984-1990 Longitudinal Study of Aging (LSOA), the 1994 Second Supplement on Aging (SOA II) and the 1994-2000 LSOA II. The 1994 SOA II served as the baseline for the LSOA II. The latter was conducted 10 years after the original LSOA in a joint effort of the National Center for Health Statistics (NCHS) and the NIA (National Institute on Aging). The sample included n = 9,447 participants; all sample persons were 70 years or older in 1995.”* |
| Setting | 5 | Describe the setting, locations, and relevant dates, including periods of recruitment, exposure, follow-up, and data collection  *-> See above* |
| Participants | 6 | (*a*) Give the eligibility criteria, and the sources and methods of selection of participants  *-> See above. A participant inclusion flow chart is provided for a better overview (Supplementary Figure 1).* |
| Variables | 7 | Clearly define all outcomes, exposures, predictors, potential confounders, and effect modifiers. Give diagnostic criteria, if applicable  *-> Page 4, section “History of relocation/moving”. Covariables and predictors are discussed in detail in the section “Covariables” on the same page.* |
| Data sources/ measurement | 8* | For each variable of interest, give sources of data and details of methods of assessment (measurement). Describe comparability of assessment methods if there is more than one group  *-> See above.* |
| Bias | 9 | Describe any efforts to address potential sources of bias  *-> Self-reported data; mentioned here. Implications including recall bias are discussed later in the discussion section.* |
| Study size | 10 | Explain how the study size was arrived at  *-> Supplementary Figure 1 – participant inclusion flowchart* |
| Quantitative variables | 11 | Explain how quantitative variables were handled in the analyses. If applicable, describe which groupings were chosen and why  *-> All quantitative variables were handled as continuous variables were provided as such by the LSOA.* |
| Statistical methods | 12 | (*a*) Describe all statistical methods, including those used to control for confounding  *-> Page 5, “Statistics”* |
|  |  | (*b*) Describe any methods used to examine subgroups and interactions  *-> Page 5, “Statistics”* |
|  |  | (*c*) Explain how missing data were addressed  *-> Participants without a complete dataset were excluded. No imputation procedures were performed.* |
|  |  | (*d*) If applicable, describe analytical methods taking account of sampling strategy  *->To account for the complex LSOAII survey design characteristics and population weights, we performed weighted survey analyses throughout the entire analysis process using Stata’s “svyset” and “svy” commands.* |
|  |  | (*e*) Describe any sensitivity analyses  *-> Not performed.* |
| Results | | |
| Participants | 13* | (a) Report numbers of individuals at each stage of study—eg numbers potentially eligible, examined for eligibility, confirmed eligible, included in the study, completing follow-up, and analysed  *-> Supplementary Figure 1 – participant inclusion flowchart* |
|  |  | (b) Give reasons for non-participation at each stage  *-> Supplementary Figure 1 – participant inclusion flowchart* |
|  |  | (c) Consider use of a flow diagram  *-> Supplementary Figure 1 – participant inclusion flowchart* |
| Descriptive data | 14* | (a) Give characteristics of study participants (eg demographic, clinical, social) and information on exposures and potential confounders  *-> Table 1. Table 1 displays sociodemographic characteristics associated with the status of moving.* |
|  |  | (b) Indicate number of participants with missing data for each variable of interest  *-> none, see above. Participants with missing data were not considered in this analysis.* |
| Outcome data | 15* | Report numbers of outcome events or summary measures  *Described in Figure 1.* |
| Main results | 16 | (*a*) Give unadjusted estimates and, if applicable, confounder-adjusted estimates and their precision (eg, 95% confidence interval). Make clear which confounders were adjusted for and why they were included  *See Figure 2. Based on a multivariate regression model adjusting for sex, age, marital status, race/ethnicity, educational level, income and health status.* |
|  |  | (*b*) Report category boundaries when continuous variables were categorized  *Not applicable.* |
|  |  | (*c*) If relevant, consider translating estimates of relative risk into absolute risk for a meaningful time period  *Not applicable.* |
| Other analyses | 17 | Report other analyses done—eg analyses of subgroups and interactions, and sensitivity analyses  *Sub-group analyses were performed and visualized in Figure 2. Sensitivity analyses were not performed..* |
| Discussion | | |
| Key results | 18 | Summarise key results with reference to study objectives  *-> Page 6, paragraphs 1 and 2. “This study used LSOA II – Wave II data to estimate the predicted prevalence of moving depending on age and other sociodemographic factors in US adults aged 70 years or older at the national level, and to explore the main reasons for moving in this population. Results may be briefly summarized as follows: The weighted percentage of participants reporting relocation within the last two years was 10.83% (9.81-11.95). The predicted prevalence increased with higher age, although no significant overall contrasts were found between females and males. Those reporting fair/poor overall health were significantly more likely to move than those with excellent health. A similar picture was found when contrasting married participants with widowed/divorced participants. Deteriorating health and the desire to go to a smaller place/apartment were the most frequently reported reasons with 35.67% (31.98-39.53) and 18.71% (15.29-22.70), respectively. More than 10% of participants who relocated since the baseline SOA II interview indicated a desire to live closer to their child/children. Participants rarely relocated because their health improved.”* |
| Limitations | 19 | Discuss limitations of the study, taking into account sources of potential bias or imprecision. Discuss both direction and magnitude of any potential bias  *-> See page 7, last paragraph: “This study has several strengths and weaknesses that warrant further consideration. The data stems from the last century (1997). However, to the best of our knowledge, this dataset has not fully exploited in the past with regard to this particular outcome. Historical perspectives may serve as a basis for foresight of population reactions and trend analyses, and may help evolve or validate trends in population behaviors (Dexter, 2006). Not analyzing this data to the fullest would result in a missed opportunity, particularly because national, epidemiological, survey-based data in this field is still scarce. Data on comorbidities was not included except for self-perceived health, which may also be a limitation. Nevertheless, the study is characterized by a fairly large sample size and the multivariate models also take non-movers into account. This study also extends previous findings by Pope and Kang (2010), who used the same dataset to compare reasons for moving in those who moved proactively with those who moved reactively. Our study goals differed in a way that we focused on multivariate regression models that explicitly took survey weights and non-movers into account. Finally, we acknowledge that reasons for moving in the LSOA II dataset were pre-coded. Questions about motivations for relocation were not open-ended Pope and Kang (2010), which may have introduced various bias to the data.”* |
| Interpretation | 20 | Give a cautious overall interpretation of results considering objectives, limitations, multiplicity of analyses, results from similar studies, and other relevant evidence  *-> See Page 8. “Results from this epidemiological analysis corroborate motivations for relocation identified in previous studies and reemphasize the complexity of relocation in older age. The estimated marginal predicted probabilities in this study highlight the complex dynamics of aging, predicted probability of moving depending on sociodemographic factors and the thereof resulting challenges with a US population that is increasingly characterized by a longer life- and short health span.”* |
| Generalisability | 21 | Discuss the generalisability (external validity) of the study results  -> n/a |
| Other information | | |
| Funding | 22 | Give the source of funding and the role of the funders for the present study and, if applicable, for the original study on which the present article is based  *The present study received no external funding.* |

## Supplementary Table 2: Bivariate associations between specific reasons for moving and the time of moving (moving in the winter months (top) *vs.* moving in the summer months (bottom)). Based on a subsample of movers with *n* = 550 participants).

| Moved in the winter months | | | |
| --- | --- | --- | --- |
| Reason | Odds ratio for bivariate logistic regression: | 95%-CI | *p*-value |
| Sample persons health deteriorated | 0.93 | 0.67-1.31 | 0.689 |
| Go to smaller place/apartment | 1.07 | 0.66-1.74 | 0.774 |
| Live closer to child | 0.98 | 0.57-1.69 | 0.948 |
| Moved to a nursing home | 0.99 | 0.55-1.77 | 0.961 |
| Spouse died | 0.98 | 0.47-2.06 | 0.969 |
| Spouse’s health deteriorated | 1.30 | 0.57-2.92 | 0.526 |
| Moved in the summer months | | | |
| Reason | Odds ratio for bivariate logistic regression: | 95%-CI | *p*-value |
| Sample persons health deteriorated | 0.93 | 0.65-1.34 | 0.713 |
| Go to smaller place/apartment | 1.00 | 0.59-1.71 | 1.000 |
| Live closer to child | 1.47 | 0.85-2.57 | 0.169 |
| Moved to a nursing home | 0.95 | 0.53-1.70 | 0.866 |
| Spouse died | 1.00 | 0.47-2.15 | 0.995 |
| Spouse’s health deteriorated | 0.93 | 0.42-2.05 | 0.861 |

# Supplementary Figures

## Supplementary Figure 1: Participant inclusion flowchart. The final sample comprised *n* = 6,091 participants, thereof *n* = 663 who moved since the LSOAII baseline interview and who were analyzed in part I of the study. For part II (analysis of marginal predicted probabilities) we excluded individuals with unknown marital status and unknown income (but otherwise available data), resulting in a final sample size of *n* = 5,444 participants. *N* = 2,406 participants were excluded for missing data on moving (implying non-participation in wave 2).

## Supplementary Figure 2: Number of reasons participants provided for moving. Based on *n* = 663 participants. 79.17% (*n* = 524) of participants provided one reason, while 5.20 % of participants (*n* = 35) provided no reason at all. A total of 91 participants (13.28%) provided two reasons.

## Supplementary Figure 3: Coefficient plot (left side) displaying regression coefficients for the regression model underlying Figure 2. The right side compares the fully adjusted logistic regression model in red color (adjusted for sex, age, marital status, race/ethnicity, educational level, income and health status) with a crude logistic regression model (age only) in blue color.

## Supplementary Figure 4: Bar chart displaying the month of moving in *n* = 663 participants.

## Supplementary Figure 5: Marginal predicted probabilities for moving in the winter months (panel A) or in the summer months (panel B) since the last LSOA II interview based on a multivariate logistic regression model (based on n = 663 unweighted observations who indicated relocation)
